# Supplementary material for: SCGN deficiency results in colitis susceptibility
Source: eLife. 2019 Oct 30;8:e49910. doi: 10.7554/eLife.49910 (PMC6839920; doi:10.7554/eLife.49910)
Supplement: Supplementary file 2. [file elife-49910-supp2.docx]

**Supplementary File 2: Scoring system for inflammation-associated histological changes in the colon (DSS)**

| **Score** | **Tissue damage in DSS colitis** | **Lamina propria inflammatory cell infiltration in DSS colitis** |  |
| --- | --- | --- | --- |
| **0** | None | Infrequent | **Involvement score**  1- 1-25% of surface  2- 26-50% of surface  3- 51-75% of surface  4- 76-100% of surface |
| **1** | Infrequent | Increased, some neutrophils |  |
| **2** | Mucosal erosions and ulcerations | Submucosal presence of inflammatory cell clusters |  |
| **3** | Extensive damage deep into the bowel wall | Transmural cell infiltrations |  |
| Total score: Sum of (Domain score x involvement score) for each domain in the scoring system | | | |
