# Supplementary material for: SCGN deficiency results in colitis susceptibility
Source: eLife. 2019 Oct 30;8:e49910. doi: 10.7554/eLife.49910 (PMC6839920; doi:10.7554/eLife.49910)
Supplement: Supplementary file 3. [file elife-49910-supp3.docx]

**Supplementary File 3: Disease activity index for colitis model**

| **Domain** | **Score** |
| --- | --- |
| Weight | 0- unchanged  1- loss of 1-5%  2- loss of 6-10%  3- loss of 11-20%  4- >20% loss |
| Stool consistency | 0- normal  2- loose stools (not watery)  4- diarrhea (liquid stool) |
| Bleeding | 0- none  2- Hematoccult positive stools  3- visible blood on stool  4- gross bleeding per rectum |
| Total score | Total score: Sum of (Domain scores) for each domain in the scoring system |
