# Supplementary material for: SCGN deficiency results in colitis susceptibility
Source: eLife. 2019 Oct 30;8:e49910. doi: 10.7554/eLife.49910 (PMC6839920; doi:10.7554/eLife.49910)
Supplement: Supplementary file 4. [file elife-49910-supp4.docx]

**Supplementary File 4: Primer sequences**

| **Gene Target** | **Forward primer sequence** | **Reverse primer sequence** |
| --- | --- | --- |
| qPCR | | |
| *Gapdh* | AGGTCGGTGTGAACGGATTTG | TGTAGACCATGTAGTTGAGGTCA |
| *Lyz* | GAGACCGAAGCACCGACTATG | CGGTTTTGACATTGTGTTCGC |
| *Muc2* | GCCTGTTTGATAGCTGCTATGTGCC | GTTCCGCCAGTCAATGCAGACAC |
| *Neurog3* | CCAAGAGCGAGTTGGCACT | CGGGCCATAGAAGCTGTGG |
| *Il1b* | GCTGAAAGCTCTCCACCTCA | AGGCCACAGGTATTTTGTCG |
| *Il6* | GTTCTCTGGGAAATCGTGGA | TTTCTGCAAGTGCATCATCG |
| *Il33* | TCCAACTCCAAGATTTCCCCG | CATGCAGTAGACATGGCAGAA |
| *Tnf* | CCCCAAAGGGATGAGAAGTT | TGGGCTACAGGCTTGTCACT |
| *Retnlb* | TTCCCACTGATAGTCCCAGG | TGCAGGAGATCGTCTTAGGC |
| *Tff3* | GCACCATACATTGGCTTGG | AGAGCCCTCTGGCTAATGCT |
| *Spdef* | CGCCTGCTGTCAGAAGAGTC | ACTGAGACCCAGCAGTGACC |
| *Scgn* | ATGGACAACGCACGCAGAAA | CCAGCTCTGTCTCTCTTATGTAACCT |
| Mutagenesis | | |
| *SCGN* | AGATGCCTCTAAAGATGGTCACATTCGGATGAAAGAGCTTG | CAAGCTCTTTCATCCGAATGTGACCATCTTTAGAGGCATCT |
| Morpholino | | |
| *scgn MO* | GCGCCATTGCTTTGCAAGAATTG | |
| *tP53 MO* | GGTTGGCAAAAGCACTGTCCATGAT | |
| CRISPR | | |
| *Scgn* exon 1 | CACCGCACGCAGAAAAACTCCAGCT | AAACAGCTGGAGTTTTTCTGCGTGC |
| *Scgn* exon 3 | CACCGAGGCCGCATACTGATGAAAG | AAACCTTTCATCAGTATGCGGCCTC |
| Genotyping | | |
| *Scgn* | GGTCGTGGTGGCTTTAACAT | CATGTCTGGCTTCCATTGTTT |
| CDX2 Cre | CGATGCAACGAGTGATGAGGTTC | GCACGTTCACCGGCATCAAC |
| *Neurog3* floxed allele | TCTCGCCTCTTCTGGCTTTC | CGGCAGATTTGAATGAGGGC |
