# Supplementary material for: SCGN deficiency results in colitis susceptibility
Source: eLife. 2019 Oct 30;8:e49910. doi: 10.7554/eLife.49910 (PMC6839920; doi:10.7554/eLife.49910)
Supplement: Supplementary file 5. [file elife-49910-supp5.docx]

| **Supplementary File 5: Key Resources Table** | | | | |
| --- | --- | --- | --- | --- |
| **Reagent type (species) or resource** | **Designation** | **Source or reference** | **Identifiers** | **Additional information** |
| gene (include species here)  (Homo sapiens) | SCGN | Open Biosystems | MHS6278-202826064 |  |
| strain, strain background (E.coli) | Competent E. coli (Stbl3 cells) | Thermo Fisher | C737303 |  |
| strain, strain background (E.coli) | Competent-DH5alpha E. coli (HIT cells) | Cedarlane | RH618 |  |
| genetic reagent (*M. musculus*) | B6.Cg-Tg(CDX2-cre)101Erf/J | Jackson laboratory | IMSR Cat# JAX:009350, RRID:IMSR_JAX:009350 | PMID: 17942902 |
| genetic reagent (*M. musculus*) | Neurog3^tm3.1Ggr^ | Gerard Gradwohl  (IGBMC) | MGI Cat# 4460259, RRID:MGI:4460259 | PMID: 20364088 |
| genetic reagent (*M. musculus*) | Scgn^Secret1^ | This paper |  | See Materials and Methods: mouse strains section |
| genetic reagent (M. musculus) | Scgn^Secret2^ | This paper |  | See Materials and Methods: mouse strains section |
| cell line (*Mus musculus*) | STC-1  intestinal neuroendocrine tumor cells | American Type Culture Collection | CRL-3254, RRID:CVCL_J405 |  |
| antibody | Anti-SNAP25 (Rabbit polyclonal) | Abcam | ab5666, RRID:AB_305033 | IF (1:150)  WB (1:1000) |
| antibody | Anti-SCGN (mouse monoclonal) | Santa Cruz biotechnology | sc-374355, RRID:AB_10989370 | IF (1:50-100)  WB (1:1000) |
| antibody | Anti-CGA (Rabbit polyclonal) | Abcam | ab15160, RRID:AB_301704 | IF (1:150) |
| antibody | Anti-CGB  (Rabbit polyclonal) | Abcam | ab12242, RRID:AB_298965 | IF (1:100) |
| antibody | Anti-TUJ1  (Mouse monoclonal) | Biolegend | 801213, RRID:AB_2728521 | IF (1:500) |
| antibody | Anti-5-HT  (Rabbit polyclonal) | Immunostar | 20080, RRID:AB_10718516 | IF (1:1000) |
| antibody | Anti-GCG  (Mouse monoclonal) | Santa Cruz Biotechnology | sc-514592, RRID:AB_2629431 | IF (1:100) |
| antibody | Anti-P84  (Mouse monoclonal) | Genetex | GTX70220, RRID:AB_372637 | WB (1:500) |
| antibody | Anti-HA  (Mouse monoclonal) | Biolegend | 901533, RRID:AB_2801249 | WB (1:500)  IP |
| antibody | Anti-Actin  (mouse monoclonal) | Sigma | A5441, RRID:AB_476744 | WB (1:5000) |
| antibody | Anti-SYP  (Rabbit monoclonal) | Abcam | ab32127, RRID:AB_2286949 | IF (1:150) |
| antibody | Lin-V450 | BD Biosciences | 51-9006958 | Flow cytometry  (5:100) |
| antibody | CD45.2-APC (Mouse monoclonal) | eBioscience | 17-0454-81, RRID:AB_469399 | Flow cytometry  (1:100) |
| antibody | NK-p46- FITC  (Rat monoclonal) | eBioscience | 11-3351-82, RRID:AB_1210843 | Flow cytometry  (2:100) |
| antibody | IL-23R-PE  (Rat monoclonal) | R&D | FAB16861P | Flow cytometry  (2:100) |
| antibody | CD4-FITC  (Rat monoclonal) | Biolegend | 100406, RRID:AB_312691 | Flow cytometry  (1:200) |
| antibody | CD11c-PE-Cy7 (Hamster monoclonal) | BD Biosciences | 558079, RRID:AB_647251 | Flow cytometry  (1:100) |
| antibody | Ly6G-eFlour-450  (Rat monoclonal) | eBioscience | 48-5931-82, RRID:AB_1548788 | Flow cytometry  (1:100) |
| antibody | CD19-V450  (Rat monoclonal) | BD Biosciences | 560375, RRID:AB_1645269 | Flow cytometry  (1:100) |
| antibody | NK1.1 FITC (Mouse monoclonal) | BD Biosciences | 553164, RRID:AB_394676 | Flow cytometry  (1:100) |
| antibody | CD25-PE  (Rat monoclonal) | eBioscience | 12-0251-81, RRID:AB_465606 | Flow cytometry  (1:100) |
| antibody | CD11b-FITC  (Rat monoclonal) | BD Biosciences | 553310, RRID:AB_394774 | Flow cytometry  (1:100) |
| antibody | Alexa Fluor 488  Goat anti-mouse) | Thermo Fisher | A-11029, RRID:AB_2534088 | IF (1:100) |
| antibody | Alexa Fluor 555  Goat anti-rabbit | Thermo Fisher | A21428, RRID:AB_2535849 | IF (1:150) |
| antibody | Anti-HA affinity matrix | Roche | Roche Cat# 11815016001, RRID:AB_390914 | IP (5µl) |
| recombinant DNA reagent | lentiCRISPR v1(plasmid) | Addgene | 49535 |  |
| recombinant DNA reagent | lentiCRISPR Scgn (plasmid) | This paper |  | pXPR_001 carrying gRNA to Scgn exon 1 |
| recombinant DNA reagent | FG9 hygro | Duckett lab, further modified by Burstein lab |  | PMID: 21706061 |
| recombinant DNA reagent | FG9 HA-SCGN | This paper |  | See Materials and Methods: cell culture section |
| recombinant DNA reagent | FG9 HA-SCGN R77H | This paper |  | See Materials and Methods: cell culture section |
| sequence-based reagent | Gapdh_F | This paper | qPCR primer | AGGTCGGTGTGAACGGATTTG |
| sequence-based reagent | Gapdh_R | This paper | qPCR primer | TGTAGACCATGTAGTTGAGGTCA |
| sequence-based reagent | Lyz_F | This paper | qPCR primer | GAGACCGAAGCACCGACTATG |
| sequence-based reagent | Lyz_R | This paper | qPCR primer | CGGTTTTGACATTGTGTTCGC |
| sequence-based reagent | Muc2_F | This paper | qPCR primer | GCCTGTTTGATAGCTGCTATGTGCC |
| sequence-based reagent | Muc2_R | This paper | qPCR primer | GTTCCGCCAGTCAATGCAGACAC |
| sequence-based reagent | Neurog3_F | This paper | qPCR primer | CCAAGAGCGAGTTGGCACT |
| sequence-based reagent | Neurog3_R | This paper | qPCR primer | CGGGCCATAGAAGCTGTGG |
| sequence-based reagent | Il1b_F | This paper | qPCR primer | GCTGAAAGCTCTCCACCTCA |
| sequence-based reagent | Il1b_R | This paper | qPCR primer | AGGCCACAGGTATTTTGTCG |
| sequence-based reagent | Il6_F | This paper | qPCR primer | GTTCTCTGGGAAATCGTGGA |
| sequence-based reagent | Il6_R | This paper | qPCR primer | TTTCTGCAAGTGCATCATCG |
| sequence-based reagent | Il33_F | This paper | qPCR primer | TCCAACTCCAAGATTTCCCCG |
| sequence-based reagent | Il33_R | This paper | qPCR primer | CATGCAGTAGACATGGCAGAA |
| sequence-based reagent | Tnf_F | This paper | qPCR primer | CCCCAAAGGGATGAGAAGTT |
| sequence-based reagent | Tnf_R | This paper | qPCR primer | TGGGCTACAGGCTTGTCACT |
| sequence-based reagent | Retnlb_F | This paper | qPCR primer | TTCCCACTGATAGTCCCAGG |
| sequence-based reagent | Retnlb_R | This paper | qPCR primer | TGCAGGAGATCGTCTTAGGC |
| sequence-based reagent | Tff3_F | This paper | qPCR primer | GCACCATACATTGGCTTGG |
| sequence-based reagent | Tff3_R | This paper | qPCR primer | AGAGCCCTCTGGCTAATGCT |
| sequence-based reagent | Spdef_F | This paper | qPCR primer | CGCCTGCTGTCAGAAGAGTC |
| sequence-based reagent | Spdef_R | This paper | qPCR primer | ACTGAGACCCAGCAGTGACC |
| sequence-based reagent | Scgn_F | This paper | qPCR primer | ATGGACAACGCACGCAGAAA |
| sequence-based reagent | Scgn_R | This paper | qPCR primer | CCAGCTCTGTCTCTCTTATGTAACCT |
| sequence-based reagent | SCGN_F | This paper | Mutagenesis primer | AGATGCCTCTAAAGATGGTCACATTCGGATGAAAGAGCTTG |
| sequence-based reagent | SCGN_R | This paper | Mutagenesis primer | CAAGCTCTTTCATCCGAATGTGACCATCTTTAGAGGCATCT |
| sequence-based reagent | Scgn_F | This paper | CRISPR guide RNA primer | CACCGCACGCAGAAAAACTCCAGCT |
| sequence-based reagent | Scgn_R | This paper | CRISPR guide RNA primer | AAACAGCTGGAGTTTTTCTGCGTGC |
| sequence-based reagent | Scgn_F | This paper | CRISPR guide RNA primer | CACCGAGGCCGCATACTGATGAAAG |
| sequence-based reagent | Scgn_R | This paper | CRISPR guide RNA primer | AAACCTTTCATCAGTATGCGGCCTC |
| sequence-based reagent | Scgn_F | This paper | PCR primer | GGTCGTGGTGGCTTTAACAT |
| sequence-based reagent | Scgn_R | This paper | PCR primer | CATGTCTGGCTTCCATTGTTT |
| sequence-based reagent | CDX2-Cre_F | Jackson labs | PCR primer | CGATGCAACGAGTGATGAGGTTC |
| sequence-based reagent | CDX2-Cre_R | Jackson labs | PCR primer | GCACGTTCACCGGCATCAAC |
| sequence-based reagent | Neurog3_F | PMID: 20364088 | PCR primer | TCTCGCCTCTTCTGGCTTTC |
| sequence-based reagent | Neurog3_R | PMID: 20364088 | PCR primer | CGGCAGATTTGAATGAGGGC |
| sequence-based reagent | scgn MO | This paper | Morpholino | GCGCCATTGCTTTGCAAGAATTG |
| sequence-based reagent | tp53 MO | This paper | Morpholino | GGTTGGCAAAAGCACTGTCCATGAT |
| commercial assay or kit | High Sensitivity GLP-1 Active ELISA Kit | Millipore | EZGLPHS-35K |  |
| commercial assay or kit | Mouse GLP-2 ELISA Kit | Crystal Chem | 81514 |  |
| commercial assay or kit | QIAamp PowerFecal DNA Kit | QIAGEN | 12830-50 |  |
| commercial assay or kit | RNeasy Mini Kit | QIAGEN | 74104 |  |
| commercial assay or kit | QuikChange Lightning Site-Directed Mutagenesis | Agilent | 210519 |  |
| chemical compound, drug | Dextran sulfate sodium salt, MW ca 40,000 | Alfa-aesar | J63606-22 |  |
| chemical compound, drug | Hoechst 33342 | Thermo Fisher | H3570 | 1:5000 |
| chemical compound, drug | Docosahexaenoic acid (DHA) | NU-CHEK | U-84-A | 100µM |
| software, algorithm | ImageJ/Fiji | National Institutes of Health | RRID:SCR_002285 | PMID: 22743772 |
| software, algorithm | RNA-seq analysis pipeline | UT Southwestern |  | See Material and Methods: RNA sequencing section |
| software, algorithm | QIIME2 | PMID: 31341288 |  | https://github.com/qiime2/q2-feature-classifier |
| software, algorithm | FLowJo v10.2 | Tree Star Inc. | RRID:SCR_008520 |  |
